# Special Issue: “G Protein-Coupled Receptor and Their Kinases in Cell Biology and Disease 2.0”

**DOI:** 10.3390/ijms232315152

**Published:** 2022-12-02

**Authors:** Alessandro Cannavo

**Affiliations:** Department of Translational Medical Sciences, Federico II University of Naples, 80131 Naples, Italy; alessandro.cannavo@unina.it

The second volume of this Special Issue, entitled “G Protein-Coupled Receptor and Their Kinases in Cell Biology and Disease 2.0”, contains nine articles, five original studies, and four reviews. This Special Issue was conceived with the purpose of advancing existing knowledge concerning the biological roles of G protein-coupled receptors (GPCRs) and their kinases, thus providing an appropriate conceptual basis for the design of novel interventions to target these receptors and their downstream and/or interacting molecules and contribute to the fight against various human diseases. GPCRs transduce different cellular signals upon binding with neurohormones, sensory signal mediators, and ions, being among the most critical receptors with crucial physiological functions [1]. Indeed, their dysfunction and hyperactivity have been systemically related to the pathogenesis and development of several disorders [1].

For instance, Nikolaev et al. [2] explored the roles of the pineal hormone melatonin and its receptors 1 (MT1) and 2 (MT2). Significantly, the authors summarized the current knowledge on the effects of these factors on cell physiology and pathology and their effects on human diseases, including type-2 diabetes, autoimmune diseases, and cancer.

Among the GPCRs, a relevant biological role has been attributed to the endothelial differentiation gene (EDG) receptors, whose activity is modulated by lysophosphatidic acid (LPA) or sphingosine-1-phosphate (S1P), which have been widely investigated [3,4]. In this sense, the review article by Solis et al. [4] explored the role of the LPA3 receptor, which has been implicated in the pathogenesis of several disorders, such as cardiac disease and cancer.

On their part, Arosio and colleagues [5] discussed the importance of sphingolipids (SLs), including S1P, and their receptors in the pathogenesis of CVDs. This study examined the correlation intercurrent between the SLs and estrogens (Es), a group of steroid compounds that function as female sex hormones, providing an accurate analysis of their impacts on the CV system in a sex-/gender-dependent manner. Significantly, Es are known to elicit several physiological activities, and their effects are mostly mediated by their interaction with the E-receptors α (ERα) and β (ERβ), which belong to the nuclear steroid hormone receptor superfamily, or the GPCR-30 (GPR30 or GPER). However, Notas and colleagues [6] demonstrated that some of the effects of E are also mediated by ERα36, a recently identified ERα variant of 36kDa, which was found to exert an inhibitory effect on NFκB-mediated inflammation in both monocytes [7] and breast cancer cells [6].

In line with the purpose of this Special Issue, Turovsky et al. [8], in their original article, investigated the mechanisms underlying the loss of rhythmicity, deregulation of the Ca2+-signaling systems, and the development of hormonal resistance to obesity. This investigation included studies on GPCRs and tyrosine kinase-coupled receptors (RTKs).

In their original report, Haji and colleagues [9] analyzed the impact of the nonsynonymous polymorphism of amino acid 64, where tryptophan can exist in place of arginine (W64R), on the expression, cellular distribution, and post-activation of the β3-adrenergic receptor (β3AR). Notably, this βAR isoform, similar to β1 and β2AR, has been reported to elicit a prominent role in the cardiovascular system, mainly in the myocardium, endothelium, and adipose tissue, modulating the cardiac function, angiogenesis, and metabolism, respectively [10,11].

In this regard, previous studies conducted in vitro demonstrated that W64R mutation generated a β3AR that was less responsive to catecholamine stimulation [11]. Therefore, several reports suggested an association between this variant and certain pathophysiological conditions (i.e., obesity) [9,11]. However, in this study, Haji et al. demonstrated that, in HEK293 cells, the WT or W64R β3AR displayed comparable biochemical properties. To be specific, these authors did not observe any differences between these two β3ARs isoforms in terms of their expression, cellular localization, and signaling activation. Thus, this study supplements other previously published studies yielding contradictory results concerning the pathogenic role of the W64R β3AR variant [11].

Notably, the discussion regarding the application of novel therapeutic approaches to target GPCR and its related factors is a Topic of extreme interest in this Special Issue. In this context, the study conducted by Kozłowska et al. [12] fits well with this Topic, since these authors assessed the effects of new agonists targeting the GPCR GPR18 in pulmonary arterial hypertension.

Analogously, in their review article, Berndt et al. [13] provided an in-depth update on the current studies regarding the GPCR-mediated regulation of the Src family kinases (SFKs), identifying the main cellular pathways that are activated and/or regulated by these kinases. SFKs are critical for cell proliferation, differentiation, and survival regulators and strongly correlated with tumor development and progression. Therefore, there is a keen interest in understanding the SFK-related signaling pathways, especially in the search for novel pharmaceutical targets against cancer.

Finally, Reichel et al. [14] explored an essential aspect underlying all the scientific research involving GPCRs and their kinases. Specifically, in their original article, these authors examined the specificity of several commercially available antibodies targeting four of the most important GRKs, including GRK2, GRK3, GRK5, and GRK6. Their results demonstrated that one of the antibodies tested did not recognize its antigen, while the others resulted in unspecific signals or cross-reactivity. Therefore, these findings suggest that the testing of an antibody should be carried out to confirm the validity of the obtained results. In addition, in this exciting study, the authors proposed a novel method, called STARPA (cost-effective simple tag-guided analysis of relative protein abundance), that enables the relative quantification of different protein isoforms via western blotting with the validated antibodies.

## Conclusions

Overall, the main goals of the first and second editions of this Special Issue have been fully achieved. For this reason, this successful Special Issue will be followed by a Topical Collection (https://www.mdpi.com/journal/ijms/topical_collections/GCPRs_Kinases) calling for further studies in this field (originals and reviews). The main aim of this Topical Collection will be to continue to provide updated information and the comprehensive elucidation of the biological roles of GPCRs and their related factors, including their kinases. This research is necessary for the development of novel research approaches that may offer benefits in the treatment of several human diseases, such as cardiovascular, neurodegenerative, and neoplastic disorders.
